# Epitaxial Growth of (−201) β-Ga_2_O_3_ on (001) Diamond Substrates

**DOI:** 10.1021/acs.cgd.3c00972

**Published:** 2023-10-16

**Authors:** Arpit Nandi, David Cherns, Indraneel Sanyal, Martin Kuball

**Affiliations:** HH Wills Physics Laboratory, University of Bristol, Tyndall Avenue, Bristol, BS8 1TL, United Kingdom

## Abstract

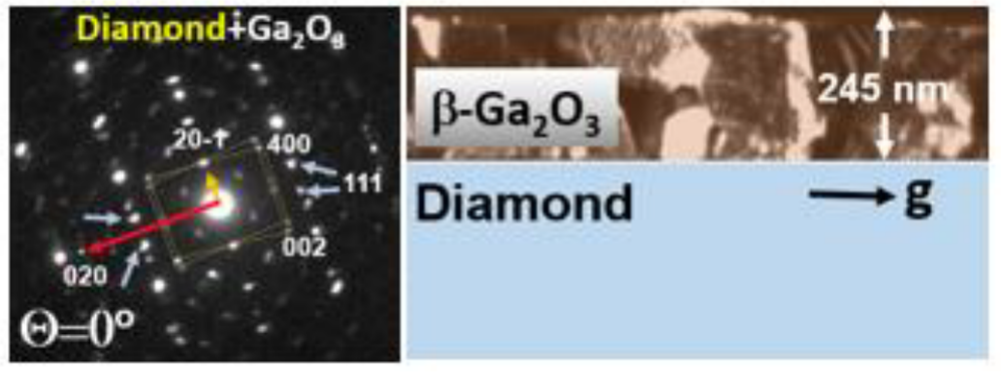

Heteroepitaxial growth of β-Ga_2_O_3_ on (001) diamond by metal–organic chemical vapor deposition (MOCVD) is reported. A detailed study was performed with Transmission Electron Microscopy (TEM) elucidating the epitaxial relation of (−201) β-Ga_2_O_3_||(001) diamond and [010]/[−13–2] β-Ga_2_O_3_ ||[110]/[1–10] diamond, with the presence of different crystallographically related epitaxial variants apparent from selected area diffraction patterns. A model explaining the arrangement of atoms along ⟨110⟩ diamond is demonstrated with a lattice mismatch of 1.03–3.66% in the perpendicular direction. Dark field imaging showed evidence of arrays of discrete defects at the boundaries between different grains. Strategies to reduce the density of defects are discussed.

## Abstract 2

Heteroepitaxial growth of β-Ga_2_O_3_ on (001) diamond by metal−organic chemical vapor deposition (MOCVD) is reported. A detailed study with Transmission Electron Microscopy (TEM) elucidating the epitaxial relation of (−201) β-Ga_2_O_3_||(001) diamond and [010]/[−13−2] β-Ga_2_O_3_ ||[110]/[1−10] diamond, with the presence of different crystallographically related epitaxial variants apparent from selected area diffraction patterns.

## Introduction

Owing to its ultrawide bandgap (4.5–4.9 eV), Ga_2_O_3_ boasts a critical electric field (*E*_c_) of 8 MV/cm, potentially giving Ga_2_O_3_ an advantage for scalable, rugged, and efficient power devices over GaN and SiC where critical fields are 2.5 times lower. As GaN and SiC technologies mature, Ga_2_O_3_ has emerged as the new favorite to provide more efficient solutions to our ever-demanding high-power commercial applications to enable a net-zero society. The availability of low-cost melt-grown substrates and the ease of n-doping offer an additional boost to its attractiveness. The Achilles’ heel for Ga_2_O_3_ is the absence of p-type doping and its poor thermal conductivity, which translates to excessive heating within high-power devices, potentially degrading device electrical performance, reliability, and lifetime. This has led to significant interest in integrating Ga_2_O_3_ with high thermal conductivity substrates to overcome the problem of the relatively low thermal conductivity of Ga_2_O_3_ itself.^1−3^ Transistors fabricated by mechanical exfoliation of Ga_2_O_3_ bonded to diamond have been demonstrated with improved heat dissipation.^4^ However, direct growth on a high thermal conductivity substrate would be preferable over a nonscalable lift-off and bonding technology by avoiding issues such as variable bonding and voids and reducing complexity in processing. It is required to have the diamond interface close to the active region of devices, and hence it becomes essential to have heteroepitaxial thin-film growth rather than bonding substrates. Recently, Low-Pressure Chemical Vapor Deposition (LPCVD) growth of continuous β-Ga_2_O_3_ films on diamond has been demonstrated; this showed the Ga_2_O_3_ layers were in (−201) orientation but provided no insight of the microstructure.^3^ Halide vapor phase epitaxially (HVPE) grown Ga_2_O_3_ reported deposition on diamond, suggesting nucleation is not trivial.^5^ Knowledge of heteroepitaxial growth on diamond is not mature compared to growth on sapphire,^6^ and a detailed epitaxial study is required. In this work, industry preferred MOCVD is used to grow phase dominant β-Ga_2_O_3_ on (001) diamond, showing epitaxial growth of (−201) β-Ga_2_O_3_ consisting of a number of different crystallographically related orientations (termed variants henceforth). MOCVD stands out among other CVD (Chemical Vapor Deposition) techniques as it demonstrates precise control over a varied range of doping densities with high electron mobility for β-Ga_2_O_3_.^7^ A model for explaining the epitaxial relationships is presented along with evidence that boundaries between grains exhibit crystalline defects. Possible ways to reduce the density of these defects are discussed.

## Experimental Details

β-Ga_2_O_3_ was grown on (001) diamond substrates provided by Element Six Technologies, using a Agnitron Agilis 100 MOCVD reactor. The typical growth range reported for β-Ga_2_O_3_ is in a window of 700–900 °C. Diamond, however, has a relatively high oxidation rate,^8^ requiring a low-temperature for the Ga_2_O_3_ growth in the oxygen-rich environment. Thus, to protect the diamond substrates; initially, a low-temperature step is needed for both the seeding and a capping layer, followed by higher temperature growth. Diamond substrates were cleaned using Acetone, IPA, and BOE, followed by DI water wash for 30 min with ultrasonication; the nitrogen (N_2_) dried substrates were then loaded to the growth chamber and kept in an N_2_ ambient environment for 30 min at room temperature, followed by a two-step growth, using a TEGa precursor and high purity oxygen (O_2_) gas (99.9999%) with argon (Ar) carrier gas: (i) a low-temperature step of 750 °C for 20 min with a VI/III ratio of 1200 to grow the seeding/capping layer, and a (ii) high-temperature step of 880 °C for 15 min with a VI/III ratio of 670 to grow the epi-layer. As diamond is prone to oxidization, O_2_ was introduced into the chamber only when the susceptor reached the growth temperature and paused during temperature ramping steps. The pressure of the growth chamber was maintained at 60 Torr throughout. Initial characterization using X-ray diffraction (XRD, Philips X’pert with Cu Kα radiation source), field emission scanning electron microscopy (FE-SEM, Zeiss Sigma HD VP), and atomic force microscopy (AFM, Bruker Edge) was performed to understand the crystallographic orientation, phase, and surface morphology of the epi-layers. To look further into epitaxial relationships, transmission electron microscopy (TEM) was used, with cross-sectional samples produced by focused ion beam (FIB) milling, with a cutting sequence of using 30 kV 30 nA, and 30 kV 3 nA followed by thinning of the lamellae at 30 kV 300 pA, 30 kV 50 pA, and finally by 5 kV 10 pA to obtain the lamellae.

## Results and Discussion

**Figure 1:**
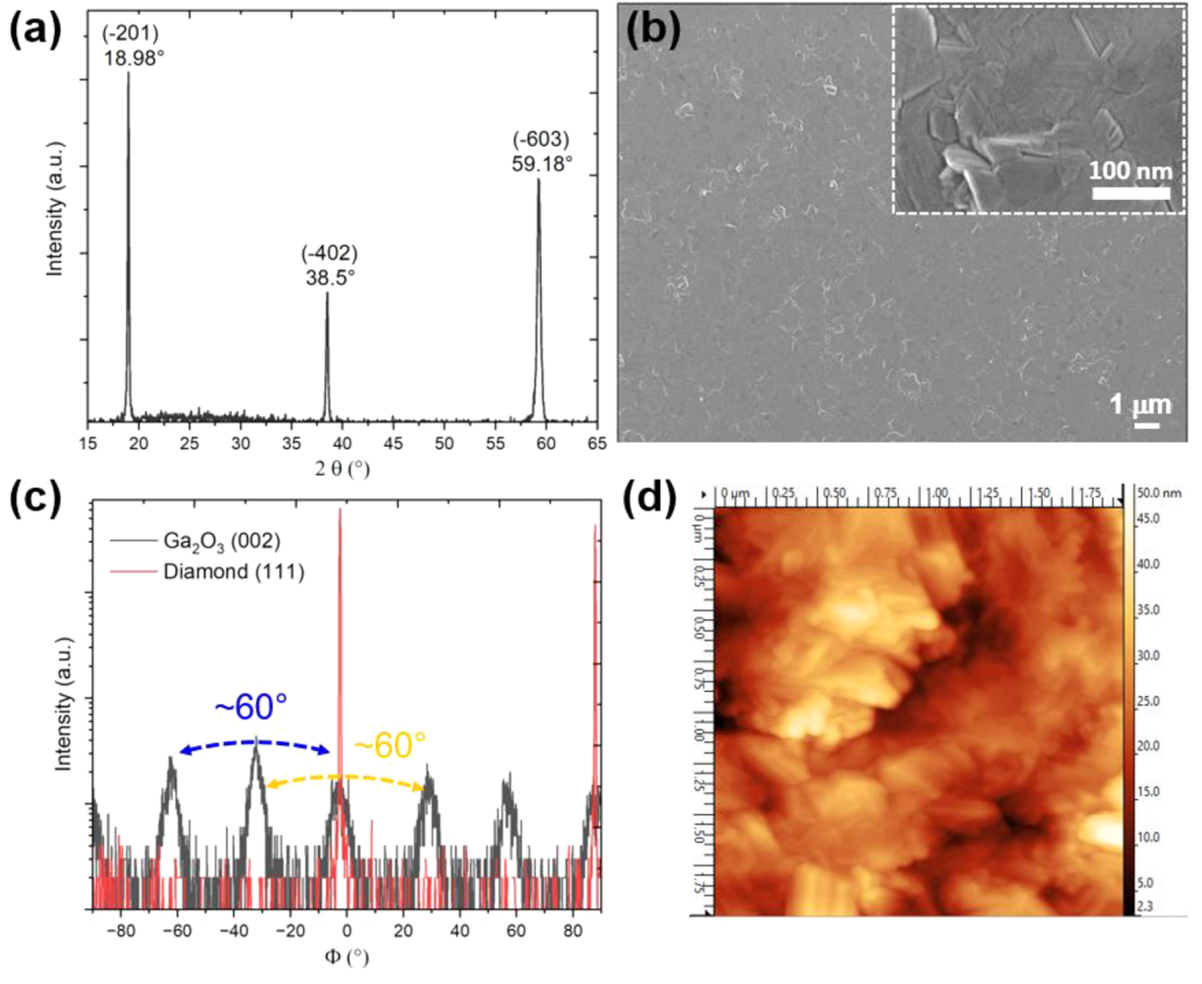
(a) High angle 2Θ–ω XRD scan from the Ga_2_O_3_ epitaxial layer grown on diamond substrates. (b) Plan view surface morphology of deposited β-Ga_2_O_3_ from FE-SEM with a low magnification and zoomed in view (inset). (c) Phi (Φ) scans of the deposited thin film showing the presence of two sets of domains ∼60° apart from each other, marked in blue and orange, respectively. (d) AFM scan of the deposited surface.

XRD measurements (Figure 1a) confirm the β-Ga_2_O_3_ (−201) out of plane orientation of the grown films.^3,9^ After optimization of both steps’ growth temperatures, a full-width half maxima (FWHM) of 2.07° was obtained from an ω-scan over the (−402) peak for an ∼0.25 μm thick film. FWHM values reported for typical heteroepitaxial growth on diamond are in ranges 1.1°–2.7°^3^ and 0.6°–2.42° for sapphire.^10,11^ Both the time and temperature of the seeding layer had an impact on the quality of the epitaxial layer. An optimum temperature of 750 °C was observed, which led to less mosaic spread of grains with dominant columnar growth as indicated by a near minimum of the FWHM at this temperature. With an increase in the seeding time from 5 to 20 min, better surface coverage of the Ga_2_O_3_ over the diamond substrate was achieved leading to more uniform films with fewer pits. The surface morphology of the thin films was investigated using FE-SEM, as shown in Figure 1b, and AFM. The inset of Figure 1b shows the zoomed in view of the grown thin film with a very characteristic pseudohexagonal structure for the monoclinic Ga_2_O_3_ with a smooth morphology. AFM scans reveal an average RMS roughness of 8.4 ± 0.8 nm across a 2 × 2 μm^2^ area as shown in Figure 1d. Figure 1c shows the phi (Φ) scans of the (002) plane of the Ga_2_O_3_ thin film and (111) diamond over a 180° range, with the arrangement of sample kept the same for both measurements. Six diffraction peaks of Ga_2_O_3_ are observed ∼30° apart. Knowing the approximate sixfold symmetry existing in (−201) β-Ga_2_O_3_^9^ these peaks can be classified arising from two sets of grains representing an epitaxial relationship in the thin film. Hence, in Figure 1c, three peaks are attributed to the presence of one of these sets separated ∼60° apart, as illustrated. Similarly, another set of peaks originate shifted by ∼30° from the previous set of grains. To further gain insight into these sets of variants, a detailed TEM analysis was performed.

**Figure 2:**
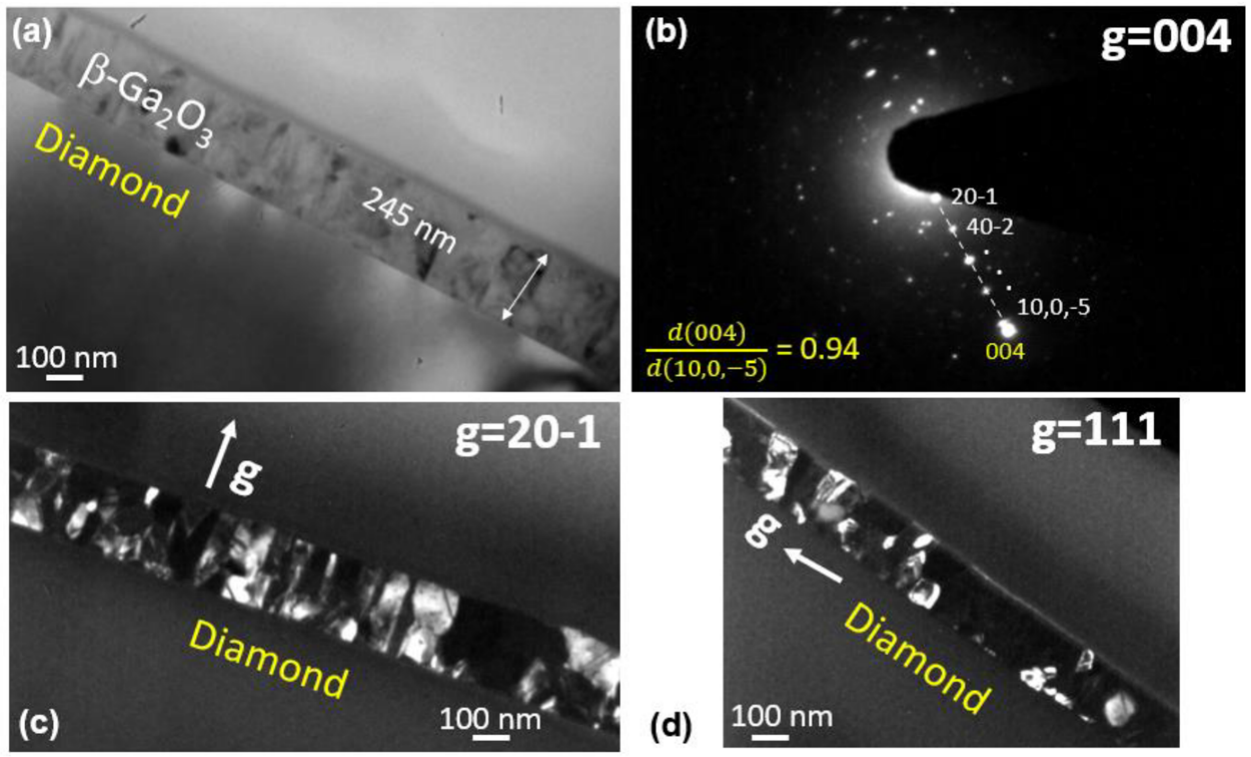
Cross-sectional TEM micrograph: (a) bright field (BF) image of the Ga_2_O_3_ epitaxial layer on the diamond substrate, (b) diffraction pattern and (c) dark field (DF) image in the 20–1 reflection, (d) DF image in 111 reflections of Ga_2_O_3_ showing a subset of set I grains in strong contrast.

Figure 2 shows the TEM images of a cross-sectional sample. Figure 2a illustrates the general bright field (BF) view of the β-Ga_2_O_3_ thin-film over the diamond substrate. Figure 2b shows a selected area electron diffraction (SAED) pattern with the Ga_2_O_3_ close to edge-on with the diamond *g* = 004 vector aligned along with the β-Ga_2_O_3_ [20–1] systematic row of reflections, confirming the parallel orientation of β-Ga_2_O_3_ (20–1) and (001) diamond planes, consistent with the XRD data. Figure 2c shows a dark field (DF) image taken in the β-Ga_2_O_3_ (20–1) reflection, indicating the columnar growth of discrete grains typically at about 50 nm in the lateral dimension. On tilting the sample by ∼1° about the axis perpendicular to [20–1], different grains showed strong contrast, illustrating slightly misoriented grains (not shown in detail).

**Figure 3:**
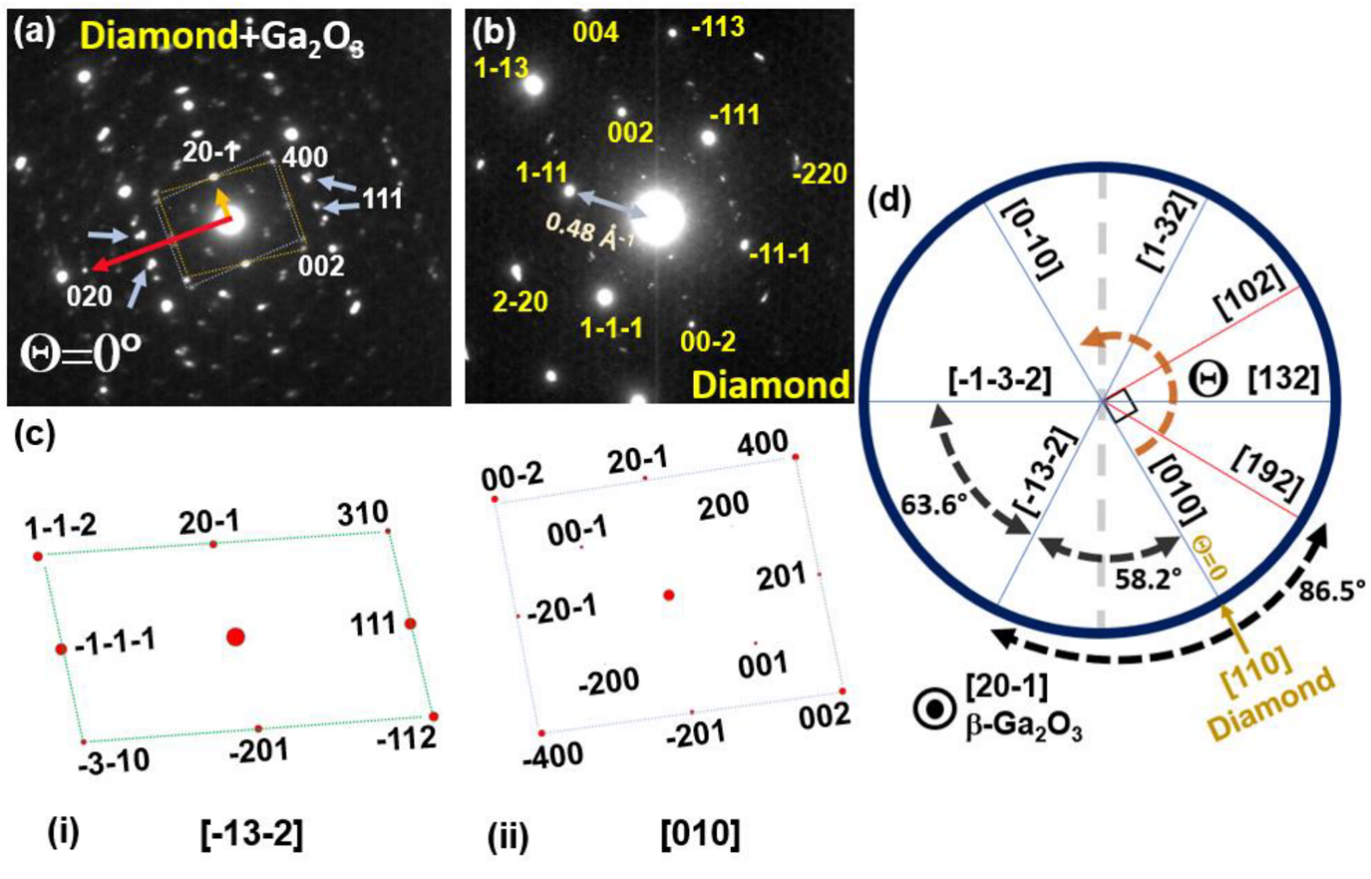
Selected area electron diffraction (SAED) pattern from (a) both set I and set II grains of β-Ga_2_O_3_ and diamond and (b) from (mainly) diamond for reference. Simulated diffraction patterns for two subsets of set I Ga_2_O_3_ variants are shown in (c) with cells corresponding to two [010] variants outlined in (a) with dotted lines; blue arrows indicating 111-type reflections for [−13–2] variants are shown in (a). (d) Schematic of the plan view (20–1) plane β-Ga_2_O_3_ with the angular relationship between various directions.

Figure 3a shows a SAED pattern from both β-Ga_2_O_3_ and diamond with the foil approximately aligned along the [110] zone axis of diamond. This illustrates the relative alignment of Ga_2_O_3_ and diamond in the interfacial plane. Figure 3 depicts the major reflections originating from only diamond. In Figure 3a, reflections from different variants of the β-Ga_2_O_3_ are visible, correlating to the [010] and [−13–2] (or the symmetry-equivalent [132]) zone axes being parallel to the [110] diamond. Two of the variants are illustrated by unit cells in Figure 3a, indicated by dotted blue and orange lines, respectively. One of these originates from the alignment of [010] β-Ga_2_O_3_ with [110] diamond (as illustrated in Figure 3c(ii)), and the other is obtained by rotating this variant by 180° about [20–1] the β-Ga_2_O_3_. These two-unit cells have a mirror relationship in edge-on planes, whose traces are parallel or perpendicular to [20–1] β-Ga_2_O_3_. Another pair of variants, due to the crystallographically equivalent [−13–2] and [132] β-Ga_2_O_3_ zone axes being parallel to [110] diamond, gives rise to the two [111]-type β-Ga_2_O_3_ reflections indicated by blue arrows. Again, these reflections come from equivalent grains with a relative 180° rotation around [20–1]. A dark field cross-sectional image (Figure 2d) highlights grains of one of these variants taken in a 111 reflection, with the interfacial plane tilted a few degrees away from edge-on condition to minimize double diffraction. Simulated diffraction patterns for the [010] and [−13–2] zone axes of β-Ga_2_O_3_ (one variant in each case) are shown obtained using PTCLab in Figure 3c(i) and (ii) for illustration.^12^Figure 3d shows the schematic plan view of the (20–1) plane with an angle between [010] and [−13–2] of 58.2°.

The four variants discussed so far relate to the alignment of the β-Ga_2_O_3_ lattice with [110] diamond, and we denote this group of grains as “set I” variants. There is also a second set of grains oriented with the [010] and [−13–2] β-Ga_2_O_3_ zone axes aligned with [1–10] diamond (perpendicular to [110] diamond) denoted in the following as “set II” variants. The diffraction spot, indexed as 020, marked by the red arrow in Figure 3a, originates from a set II variant since the rotation of 90° between the [110] and [1–10] diamond means that the [010] and [−13–2] axes from β-Ga_2_O_3_ are now horizontal. For the [010] oriented grains, this means that the 020 planes are edge-on, giving relatively strong diffraction with the electron beam directed along [102] β-Ga_2_O_3_ (Figure 3d). In contrast, for the set II [−13–2] oriented grains, there is no strong reflection along [−13–2], the nearest strong reflection (very close to the 020 reflection, see discussion later) being a 5–12 reflection whose planes are edge-on at a rotation of 86.5° from the [−13–2] direction (at a [192] β-Ga_2_O_3_ pole, Figure 3d). It is worth mentioning at this point that all of the diffraction spots obtained in the SAED shown in Figure 3 can be indexed as β-Ga_2_O_3_ set I or set II variants, but only the planes of interest have been highlighted and labeled.

A rotation θ (Figure 3d) of near 60° and 120° around [20–1] β-Ga_2_O_3_ from the orientation in Figure 3 should bring either [010] or [−13–2] zone axes for set I variants within ±1.8° to the beam direction, whereas rotations of 30° and 90° should do the same for set II variants and therefore we expect similar diffraction patterns for the β-Ga_2_O_3_ to occur at 30° rotation intervals, albeit that the intensities of the spots for the different variants may be reduced by being slightly off the strongest diffraction conditions.

**Figure 4:**
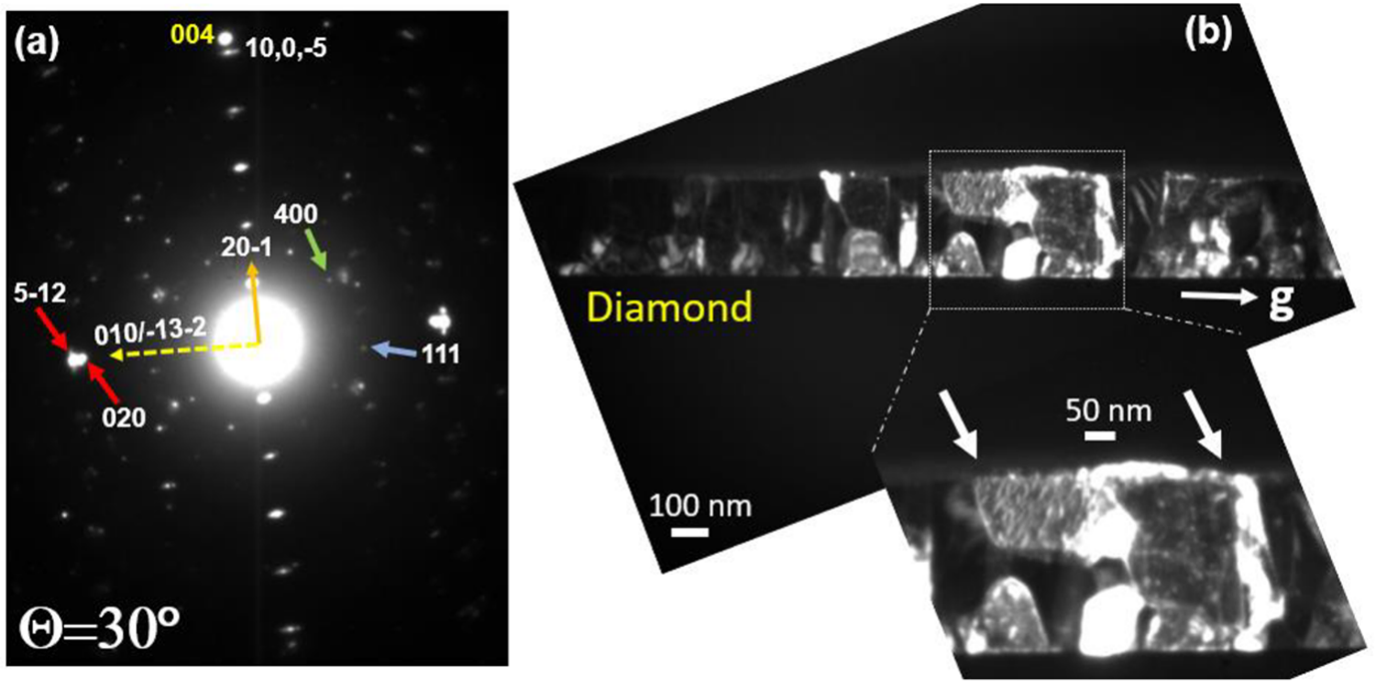
(a) Selected area diffraction pattern at θ = 30° rotation from [110] diamond, showing β-Ga_2_O_3_ reflections from set I and set II variants. (b) DF image taken with *g* = 020/5–12 doublet (indicated by red arrows) showing contrast from set I grains; zoom-in image of a section suggesting defects at boundaries between set I grains.

Figure 4a shows a pattern at θ = 30° rotation from Figure 3a. Compared with Figure 3a, the only diamond reflection visible is the 004 reflection, as we are no longer at a major diamond zone axis. However, the variant patterns illustrated in Figure 3a are again visible in Figure 4a, albeit relatively weakly, and relate to set II variants as noted above. The pattern also shows a fairly strong doublet spot, as labeled by red arrows. This consists of a 020 spot from [−13–2] variants from set I, with the (020) planes about 1.8° away from being edge-on. The second spot is a 5–12 reflection from the [010] variants from set I, with (5–12) planes also tilted by 1.8° from edge-on but in the opposite direction. Therefore, the significance of the 020/5–12 doublet is that it should give appreciable contrast in dark field images and diffraction from both [010] and [−13–2] variants, allowing us to both confirm the presence of rotational domains and image the nature of the boundaries between them. A dark field image taken in this doublet reflection is shown in Figure 4b, which shows networks of discrete defects, which we assume are most likely at set I variant-to-variant grain boundaries.

**Figure 5:**
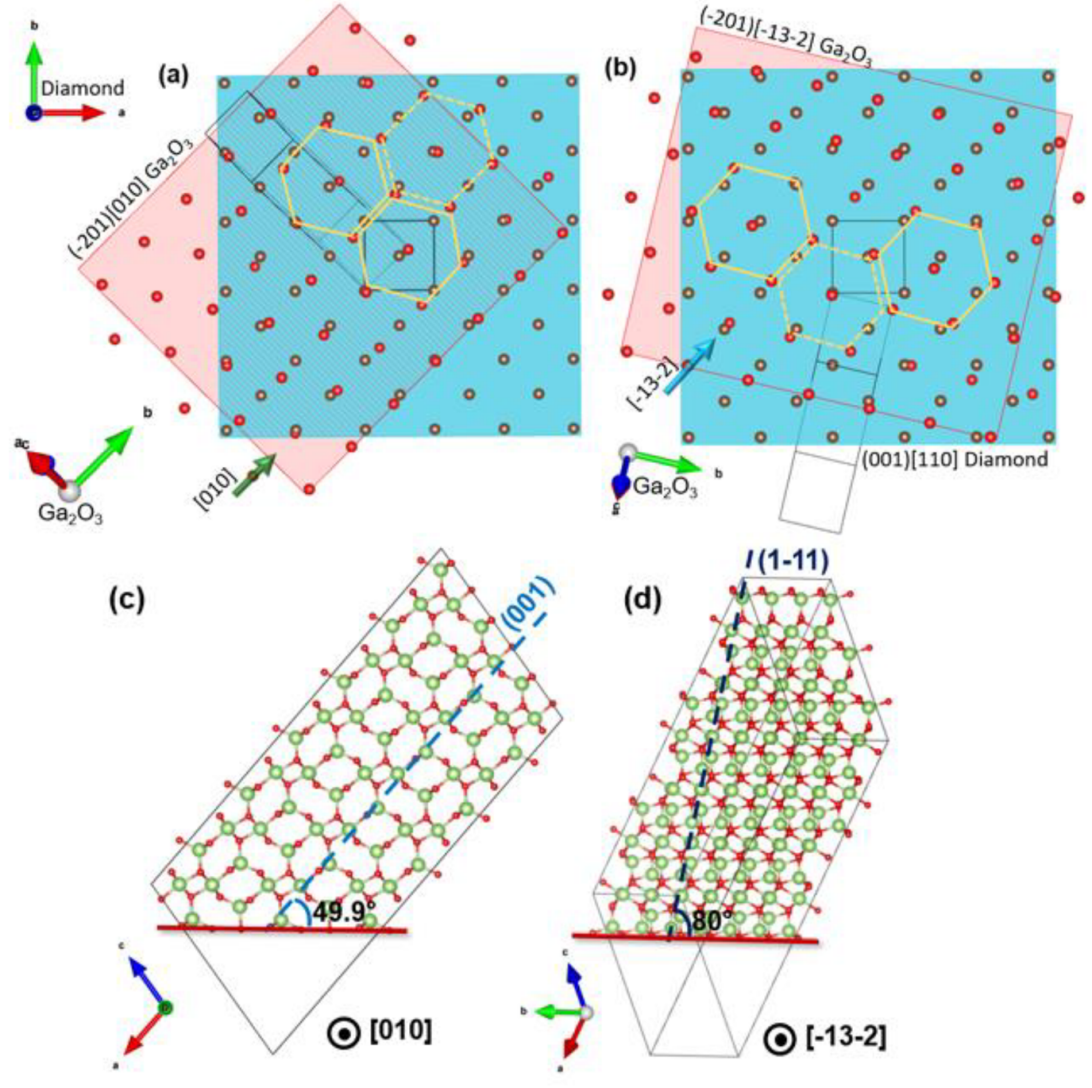
Visual model of (−201) β-Ga_2_O_3_ on diamond for one set of variants from the top view. The red spheres denote the oxygen atoms on the (20–1) plane of Ga_2_O_3_ arranged in askew hexagonal pattern over the carbon atoms (brown) of the cubic diamond lattice. (a) Shows arrangement of the [010] direction of β-Ga_2_O_3_ with [110] diamond while (b) shows the [−13–2] direction of (−201)β-Ga_2_O_3_ with [110] diamond. While (c) and (d) depict the respective directions cross-sectional view (generated with Visualization for Electronic and Structural Analysis (VESTA) software^15^). The second set of variants is obtained by aligning [010] and [−13–2] directions of β-Ga_2_O_3_ with [1–10] diamond.

The results in Figures 2–4 show that there is an epitaxial relationship for (−201) β-Ga_2_O_3_ on (001) diamond with [010]/[−13–2] β-Ga_2_O_3_ ||[110] diamond with an equivalent set of variants rotated by 90°. A model showing the plan view arrangement of the interfacial planes of atoms along [010] and [−13–2] for β-Ga_2_O_3_ when aligned with the [110] diamond (set I) is shown in Figure 5a and b, respectively. Along with the atom alignment along [110] diamond, the atom rows appear relatively well matched in spacing along the perpendicular direction, i.e. [1–10]. However, it is essential to note that the close coincidence of the atom rows along [110] diamond is merely shown for clarity since there may be relative translations of the two lattices parallel and perpendicular to this direction. The lattice mismatch in the [1–10] direction was calculated to be 1.03% and 3.66% for [010] and [−13–2] of β-Ga_2_O_3_, respectively.

As far as the crystalline perfection of the β-Ga_2_O_3_ on diamond is concerned, grain misorientations are not random, and high-angle grain boundaries with high densities of defects may therefore be avoided. The results suggest that our films contain eight distinguishable epitaxial variants (four each from set I and set II, taking symmetry into account). Grain boundaries between the different variants will include defect-free boundaries where neighboring grains are of the same variant, twin boundaries where there is a twin relationship and other more complex boundaries where [010] and [−13–2] variants of one set meet, or where boundaries of set I variants meet those of set II. It is desirable to have fewer boundaries either by reducing the grain nucleation density or by promoting the growth of one set of variants over the other, or indeed growth of a single variant. A previous study of β-Ga_2_O_3_ growth on c-plane sapphire^6^ shows unit cells similar to the variants reported here. Therefore, this study shows that β-Ga_2_O_3_ epitaxial growth on diamond in some form is comparable to that on sapphire. However, β-Ga_2_O_3_ growth on the cubic face of the diamond produces two sets of epitaxial variants compared with only one set for growth on the hexagonal sapphire surface. The use of off-cut substrates has been effective for reducing the multiplicity of variants in the epitaxial growth of β-Ga_2_O_3_ on c-plane sapphire as well as reducing defects in homoepitaxial growth of β-Ga_2_O_3_;^13,14^ this suggests off-cut substrates could be effective for reducing defects in the epitaxial growth of β-Ga_2_O_3_ on diamond substrates.

## Conclusion

In summary, a two-step process for MOCVD grown epi-layers of (−201) β-Ga_2_O_3_ over single crystalline diamond substrates was demonstrated; the samples grown were studied using XRD, SEM, and cross-sectional TEM. TEM experiments with the sample tilted in different directions revealed two sets of epitaxially related grain variants, denoted as sets I and II, with each set having four distinguishable variants. A growth orientation relationship of [010]/[−13–2] _film_||[110]_substrate_ and [010]/[−13–2]_film_||[1–10]_substrate_ was confirmed with a corresponding lattice mismatch between 1.03 and 3.66% along [1–10] diamond. The presence of such sets of grains can give rise to defects at the grain boundaries between different directions, hampering the formation of a single-crystalline thin film. Using [110] diamond off-cut substrates may help reduce the multiplicity of variants and improve epi-layer growth.
